# The use of purple carrot powder in the diet of laying quails improved some egg quality characteristics, including antioxidant capacity

**DOI:** 10.1007/s11250-023-03636-x

**Published:** 2023-05-23

**Authors:** Ainhoa Sarmiento-Garcia, Osman Olgun, Gözde Kilinç, Behlül Sevim, Seyit Ahmet Gökmen

**Affiliations:** 1 Área de Producción Animal, Departamento de Construcción Y Agronomía, Facultad de Ciencias Agrarias Y Ambientales, Universidad de Salamanca, 37007 Salamanca, Spain; 2 Department of Animal Science, Faculty of Agriculture, Selcuk University, 42130 Selcuklu Konya, Turkey; 3 Department of Food Processing, Suluova Vocational Schools, Amasya University, 05500 Amasya, Turkey; 4 Eskil Vocational School, Aksaray University, 68800 Aksaray, Turkey

**Keywords:** Antioxidant capacity, Circular economy, Egg production, Egg quality, Purple carrot

## Abstract

The goal of the current experiment was to investigate the effect of dietary concentrations of purple carrot powder (PCP) on performance, egg production, egg quality, and the antioxidant capacity of the yolk in laying quails. A total of one hundred and fifty 22-week-old Japanese laying quails were allotted to 5 dietary treatments each with 6 replicates of 5 quails. Quails were allocated to five dietary treatments (0, 0.1, 0.2, 0.3, and 0.4%) with PCP addition at an increasing level from 0 to 4000 mg/kg diet respectively, which were fed *ad-libitum* throughout the duration of the experiment. No differences were detected between dietary treatments for any of the performance parameters or egg production. Eggshell weight and eggshell thickness (*P* < 0.05) were linearly affected by PCP dietary, reaching maximum levels at 0.4% of PCP supplementation, while the percentage of damaged egg and egg-breaking strength remained similar for all experimental groups (*P* < 0.05). Quails receiving PCP diets showed a yellowness (b*) (*P* < 0.05) egg yolk color than those fed the control diet, without affecting the rest of the color parameters and egg internal quality. Increasing PCP levels in diets reduced linearly yolk TBARS (*P* < 0.01) and increased linearly DPPH (*P* < 0.01). The addition of PCP, a safe and readily available agricultural by-product, as a component of the diet of laying quail was effective without adversely affecting quail production. Moreover, the inclusion of PCP in the diet might benefit laying quails’ eggs by improving some quality traits and enhancing the yolk’s antioxidant capacity, which could improve their shelf-life and acceptability.

## Introduction

Many consumers in several countries such as Türkiye, Egypt, and Indonesia prefer quail eggs for their various benefits. Improving poultry hens’ productivity and egg quality by using natural botanical by-products is a very critical subject nowadays in light of the scarcity and the rising cost of conventional diet ingredients (El-Sabrout et al., 2022, 2023; El-Saadany et al., 2022). The sensory and nutritional quality of eggs are increasingly a concern to consumers, and attributes such as yolk color are important to purchase criteria (Panaite et al., 2021). In this manner, nutrition can play an important role to modify poultry diet composition (Gül et al., 2022; El-Sabrout et al., 2023) and, in particular, the use of carotenoids in the diet has been a popular practice to modify that (Díaz-Gómez et al., 2017; Kljak et al., 2021a, b; Panaite et al., 2021; Maia et al 2022; Meléndez-Martínez et al., 2022). Carotenoids are isoprenoid-based molecules distributed in all photosynthetic organisms (Pérez-Gálvez et al., 2020) widely known for the pigmenting action of their xanthophylls, antioxidants, and pro-vitamin A (de Souza et al., 2019). For many years, carotenoids have been widely used as pigments in poultry diets to modify egg yolk color (Hammershøj et al., 2010), and these changes depend on the concentration, source, and ratio of carotenoids (Kljak et al., 2021b). The importance of carotenoids is not limited to changes in egg or meat quality, supplementing poultry with dietary carotenoids may enhance productive performance and avian health (Langi et al., 2018). Carotenoids are an important biomarker linked to declines in certain degenerative disorders (such as cancer of the lung, gastrointestinal tract, pancreas, or breast). It is due to that they are involved in multifactorial pathways (Pérez-Gálvez et al., 2020; Meléndez-Martínez et al., 2022), such as scavenging free radicals or stimulating the generation of antioxidant enzymes (Nabi et al., 2020). However, information on the feed’s carotenoid content is limited compared to food. Only those feeds high in synthetic carotenoids, which are needed for the pigmentation of animal products, have been thoroughly evaluated (Meléndez-Martínez et al., 2022). Even though a wide number of synthetic pigments are available on the market, consumers are becoming more concerned about the consumption of synthetic additives in food and feed and their impact on health, which has led to an increasing interest in natural alternatives (Hammershøj et al., 2010; Langi et al., 2018; Kljak et al., 2021a,b; Meléndez-Martínez et al., 2022). In addition, the use of synthetic additives leads to an increase in the overall cost of egg production and adversely affects the environmental footprint of animal feed production. For these reasons, many researchers have focused their efforts on identifying the presence of carotenoids in vegetables (Hammershøj et al., 2010; Langi et al., 2018; Kljak et al., 2021a, b; Meléndez-Martínez et al., 2022).

The carrot (*Daucus carota L.*), belonging to the family *Apiaceae*, is a root vegetable rich in fiber, minerals, polyphenols carbohydrates, and antioxidant flavonoids whose content depends on the varieties studied (Panaite et al., 2021; Yusuf et al., 2021). There is growing interested and acceptance among consumers for special carrots of different colors (Hammershøj et al., 2010; Yusuf et al., 2021). Carrots of low quality (22% of total production) are discarded for various reasons such as root size, deformation, breakage, or disease. With the increasing production of different colored varieties, the amount of rejected carrots of these special carrots is increasing as well, so these species offer low economic value to be used as animal feed (Hammershøj et al., 2010; Titcomb et al., 2019). Among that, purple carrots (*Daucus carota*. ssp. s*ativus* var. *atrorubens* Alef.) are an essential variety of carrots due to their antioxidant content (such as anthocyanins and carotenoids), being responsible for their typical color (Yusuf et al., 2021).

In recent years, Japanese quails (*Coturnix coturnix Japonica*) production has increased worldwide, due to its easy handling and the growing popularity of its eggs and meat (Sarmiento-García et al., 2022). In contrast to traditional poultry species, fewer studies have been conducted on the nutrition of this species. Above the aforementioned information, it would be interesting to know whether the bioactive substances in purple carrots can be assimilated by the avian´s metabolism and, subsequently, can be transferred to the egg. This would provide important information on the role of purple carrots in quail feeding. Therefore, this research aimed to establish the effects of purple carrots at different concentrations on performance, egg quality parameters, yolk color, and antioxidant capacity.

## Materials and methods

### Ethical approval

The current research was carried out on farm animals, so no special certification was required for breeding laboratory animals. Nevertheless, criteria specified by European policy for the protection of animals (EPCEU, 2010) were followed during the experimental period.

### Experimental design

The whole experiment was conducted on 150 female Japanese quails with a similar body weight (259.90 ± 9.37 g) and 22 weeks of age. The study was carried out by a completely randomized design at a local indoor farm in Selçuklu, Konya, Türkiye (38°1′36″, 32°30′45″) for 70 days. The trial was performed with 5 experimental groups of 6 replicates, each containing 5 female quails. The quails were randomly assigned into five equal, clean, well-aired, and sanitized battery cages (30 cm wide and 45 cm long), presenting identical features. A temperature of 22 ± 2.0 °C and a 16-h lighting program were provided in each pen. All pens were provided with individual feeders and drinkers to enable ad libitum feed intake.

For the experimental treatments, the supplementation was done with PCP which was provided by a local market (Dağinciri Ltd. Şti., Aydın, Turkey). The method proposed by Singleton and Rossi (1965) was used to determine the total phenolic content and reducing power (%). Moreover, the half maximal inhibitory concentration (IC50) was calculated according to the method proposed by Singh et al. (2002) (Table 1). The PCP was added to the basal diet at inclusion levels of 0, 0.1, 0.2, 0.3, and 0.4% to design the treatments. PCP addition levels (< 0.4%) did not alter the nutrient content of the basal diet; thus, all diets can be regarded as isocaloric and isonitrogenous. The basal diet was designed according to the National Research Council (1994) recommendations to meet requirements in layer quails. The chemical composition of the PCP and the basal diet were analyzed according to AOAC (2006) proceedings and are reported in Tables 1 and 2, respectively.

**Table 1:** Chemical composition, reducing power, total phenolic content and the half maximal inhibitory concentration of purple carrot powder

Parameters	Level
Crude protein	66.90
Crude ash	64.60
Crude fat	10.80
Crude cellulose	190.80
Calcium	2.50
DPPH (% reducing)	11.80
Total phenolic content (g/kg GAE)	5.60
IC_50_	7674.74

Chemical composition of purple carrot powder was expressed as g/kg. *DPPH* 2,2-diphenyl-1-picrylhydrazyl, *GAE* Gallic acid equivalent, *IC*_*50*_ The half maximal inhibitory concentration

**Table 2:** Basal diet and nutrient composition

Ingredient	g/kg	Nutrient composition	g/kg
Corn	570.0	Metabolizable energy (kcal ME/kg)	2902
Soybean meal (460 g/kg CP)	261.0	Crude protein	199.9
Meat-bone meal (450 g/kg CP)	27.6	Crude fat	73.5
Full-fat soybean	65.0	Crude cellulose	39.7
Limestone	52.0	Moisture	125.2
Sunflowers oil	17.1	Calcium	25.0
Salt	3.0	Available phosphorus	3.5
Premix^1^	2.5	Lysine	10.6
DL methionine	1.8	Methionine	4.6
Total	1000.0	Cystine	4.1
		Methionine + Cystine	8.7

*CP* crude protein. ^1^Premix provides 80 mg manganese (manganese oxide), 60 mg iron (iron carbonate), 5 mg copper (copper sulfate pentahydrate), 1 mg iodine, 0.15 mg selenium, 8800 IU vitamin A (trans-retinol acetate), 2200 IU vitamin D3 (cholecalciferol), 11 mg vitamin E (tocopherol), 44 mg nicotinic acid, 8.8 mg Cal-D-Pan, 4.4 mg Vitamin B2 (riboflavin), 2.5 mg thiamine, 6.6 mg vitamin B12 (cyanocobalamin), 1 mg folic acid, 0.11 mg biotin, 220 mg choline to per kg of diet

### Determination of productive performance parameters

Upon arrival at the facility, the quails were randomly distributed according to the experimental diets. For determining body weight changes, each quail was weighed (± 0.01 g) at both the beginning and end of the assay and the differences in body weight (± 0.01 g) were determined. Feed intake was estimated by subtracting the quantity supplied from the amount of feed left over in the feeder for each experimental unit as shown by Olgun et al. (2022). The calculated result was divided by the number of quails and by the days the quails fed. The feed intake is expressed in g/bird/day. Average body weight gain (g/day) was determined by subtracting initial body weight (g) from final body weight (g) over the study period.

On each day of the study, eggs were collected at the same hour (10:00 a.m.). For determining egg production, the recorded number of eggs per day was divided by the total number of birds and multiplied by 100. The result was given as a percentage (%). For the determination of egg weight, every egg obtained in the last 3 days of the research was weighed with a high precision balance (± 0.01 g). From these values, the egg mass was estimated using the next Eq. (1):

1 $$Egg\;mass=\frac{\left(egg\;production\;\left(\%\right)\times egg\;weight\;\left(g\right)\right)}{100}$$

Lastly, the feed conversion ratio was determined following to the next Eq. (2):

2 $$Feed\;conversion\;ratio=\frac{feed\;intake\;(g\;feed)}{egg\;mass\;(g\;egg)}$$

### Determination of egg quality parameters

The following methods were carried out at the Egg Quality Laboratory (Faculty of Agriculture, Selcuk University, Konya, Türkiye). All the eggs collected in the trial’s last 3 days were tested for internal and external quality standards at environmental temperature. Broken, damaged, and cracked eggs were recorded over the experiment and determined as a percentage of the total number of eggs (*n* = 400). A cantilever system is used to determine breaking strength by applying a growing pressure on the wide pole of the shell using the Egg Force Reader (Orka Food Technology Ltd., Ramat Hasharon, Israel). The three sections (equator, blunt, and pointed parts) values of the eggshells were determined using a digital micrometer (Mitutoyo, 0, 01 mm, Japanese). These results were used to determine the eggshell thickness (µm).

For evaluation of the internal quality of the eggs, they were cracked on a cleaned glass surface and the shell residues were discarded. For the determination of eggshell weight, the eggs were pre-dried at room temperature for 3 days. The shell weight was determined as a function of egg weight. Subsequently, the albumen was removed from the yolk. Albumen and yolk heights were determined with a height gage, and length and width with a 0.01 mm digital caliper (Mitutoyo, Japan). The above values were used to determine the albumen index (3):

3 $$Albumen\;index=\frac{Albumen\;height\;(mm)}{\frac{Albumen\;width+Albumen\;length}2(mm)}x100$$

Yolk index was determined according to the next Eq. (4)

4 $$Yolk\;index=\frac{Height\;of\;yolk}{Diameter\;of\;yolk\;(mm)}x100.$$

Lastly, Haugh unit was obtained from the egg weight and albumen height data in accordance with the Eq. (5) provided by Stadelman and Cotterill (1995).

5 $$Haugh\;unit=100\;x\;\log(albumen\;height+7.57-1.7x{EW}^{0.37})$$

For colorimetric analysis, the samples were placed in Petri dishes which allowed for maintaining the integrity of the egg yolks. To determine L* (lightness), a* (redness), and b* (yellowness) values, egg yolks were tested with a pre-calibrated Konica Minolta digital colorimeter (Minolta Chroma Meter CR 400 (Minolta Co., Osaka, Japan) as described Titcomb et al. (2019).

### Determination of TBARs and DPPH in the yolk

To determine lipid peroxidation, the modified yolk thiobarbituric acid reactive substances (TBARS) assay proposed by Kilic and Richards (2003) and Sarmiento-García et al. (2021) was performed three times for each sample (*n* = 100). Two grams of the yolk were collected and mixed with 12 ml of the trichloroacetic acid (TCA) solution. The solution was blended for 20 s in ultraturrax (IKA, USA) and it was filtrated. The mixture was poured into tubes, and 3 ml of the thiobarbituric acid (TBA) solution (0.02 M) was incorporated. The mixture was boiled in a water bath for 40 min to acquire a pink color and centrifuged for 5 min at 2000 rpm. The supernatant was spectrophotometrically determined at a 530 nm wavelength in a spectrophotometer (Perkin Elmer, USA) versus a blank consisting of 1 mL TCA extraction solution and 1 mL TBA solution. The TBARS were estimated from a standard curve of malondialdehyde, used for preparing the reference curve. TBA (6) was determined as µmol MDA/g yolk according to the next Eq. (6):

6 $$TBA\;Value=\frac{(absorbance/kx2/1000)x0.8}{sample\;weight}X100$$

The antioxidant capacity of hydrolysates was tested on the radical scavenging effect 2,2-diphenyl-1-picrylhydrazyl (DPPH)-free radical activity following the adapted method proposed by Sacchetti et al. (2005). Two grams of yolk were isolated and diluted in 25 ml of 95% methanol, and then the removal procedure was carried out in an ultrasonic bath for 20 min. The mixture was filtrated and collected in 0.1-ml glass tubes. 2.9 mL of DPPH solution (100 mL of methanol (100%) + 0.0025 g of DPPH (97%) was incorporated into the solution, then it was mixed for 25 s in a vortex. Once the mix has been allowed to stand at temperature for 30 min, the absorbance was measured using a spectrophotometer (Perkin Elmer precisely UV/VIS Spectrometer) at 517 nm wavelength. Control was performed similarly, with 95% ethanol replacing the sample solution. To determine the average value, each experiment was carried out in triplicate. Equation (7) described by Sacchetti et al. (2005) was used to calculate DPPH values:

$$DPPH\;values=\frac{\lbrack(Control\;absorbance-Sample\;absorbance)}{Control\;Absorbance}x100$$

### Statistical analysis

Data were analyzed by one-way ANOVA using the SPSS 22.0 software package (SPSS Inc., Chicago, IL, USA), using the cage means as an experimental unit. A probability value of *P* < 0.05 was considered statistically significant. Orthogonal polynomial contrasts were used to assess the significance of linear and quadratic models to describe the response of the dependent variable to a rising PCP level.

## Results

In all dietary treatments, no mortality or symptoms of the disease were observed at the end of the trial. The findings in Table 3 demonstrated that feeding different levels of PCP not affected (*P* > 0.05) performance parameters of laying quails (in terms of initial body weight, final body weight, body weight gain, feed intake, and feed conversion ratio). Likewise, there have been no significant differences (*P* > 0.05) in egg production (including egg production, egg weight, or egg mass). The minimum and maximum values for performance parameters and egg production were as follows: initial body weight (258.0–267.5 g), final body weight (271.1–288.4 g), body weight change (13.1–20.9 g), egg production (91.07–92.43%), egg weight (12.46–13.05 g), egg mass (11.36–12.00 g/day/quail), feed intake (34.07–35.77 g/day/quail), and feed conversion ratio (2.94–3.01 g feed/g egg).

**Table 3:** Influence of dietary supplementation with purple carrot powder (PCP) on performance and egg production in laying quails

Parameters	Purple carrot powder (%)					S.E.M*	P	L	Q
	0	0.1	0.2	0.3	0.4				
Initial body weight (g)	267.5	258.0	258.2	258.8	265.3	4.68	0.485	0.819	0.081
Final body weight (g)	288.4	271.1	271.5	277.8	283.1	2.71	0.190	0.829	0.057
Body weight gain (g)	20.9	13.1	13.3	19.0	17.8	3.12	0.387	0.963	0.147
Egg production (%)	91.43	91.19	92.00	91.07	92.43	0.774	0.722	0.462	0.630
Egg weight (g)	13.05	12.46	13.05	13.01	12.92	0.248	0.459	0.712	0.703
Egg mass (g/day/quail)	11.93	11.36	12.00	11.86	11.95	0.240	0.352	0.493	0.574
Feed intake (g/day/quail)	35.56	34.07	35.24	35.47	35.77	0.766	0.578	0.468	0.379
FCR (g feed/g egg)	2.99	3.01	2.94	2.99	3.00	0.076	0.973	0.997	0.752

*FCR* feed conversion ratio, *S.E.M*.* standard error means, *L* linear effect, *Q* quadratic effect

As can be observed in Table 4, eggshell weight (*P* < 0.01) and eggshell thickness (*P* < 0.01) were linearly affected by the PCP diet. Similar trends were observed for both parameters, as PCP levels increased these values grew, to reach a maximum when quails were fed 0.4% PCP (8.90% and 250.3 µm, respectively). No differences (*P* > 0.05) between experimental diets regarding damaged eggs and egg-breaking strength were found.

**Table 4:** Effect of purple carrot powder diet on egg external quality in laying quails

Parameters	Purple carrot powder (%)					S.E.M*	P	L	Q
	0	0.1	0.2	0.3	0.4				
Damaged egg (%)	0.52	0.14	0.15	0.13	0.49	0.198	0.469	0.920	0.076
Egg-breaking strength (kg)	1.52	1.45	1.44	1.48	1.55	0.060	0.688	0.665	0.165
Eggshell weight (%)	8.22^b^	8.14^b^	8.25^b^	8.46^ab^	8.90^a^	0.142	0.021	0.009	0.142
Eggshell thickness (µm)	238.1^b^	236.0^b^	244.2^ab^	245.2^ab^	250.3^a^	6.83	0.027	0.003	0.540

S.E.M.* standard error means, *L* linear effect, *Q* quadratic effect. ^a,b^Means with different superscripts in the same row were significantly different (*P* < 0.05)

Table 5 demonstrates that feeding at different levels of PCP does not negatively affect egg quality in terms of the albumen index, yolk index, and Haugh unit (*P* > 0.05). Those values ranged between 2.24 and 2.50 for the albumen index, 43.74 to 46.82 for the yolk index, and 85.19 to 87.38 for the Haugh unit. The yolk color was expressed as lightness (L ∗), redness (a ∗), and yellowness (b ∗) values. Supplementation with PCP significantly increased the b* value (yellowness) in all supplemented groups regarding the control group (31.96), implying redder yolk than the control group (*P* < 0.05). However, the carrot-supplemented treatment did not affect the rest of the color parameters. The L*(lightness) and a*(redness) values of the control were similar to that of all PCP-supplemented treatments.

**Table 5:** Effect of addition of purple carrot powder in the diet on egg internal quality in laying quails

Parameters	Purple carrot powder (%)					S.E.M*	P	L	Q
	0	0.1	0.2	0.3	0.4				
Albumen index	2.24	2.31	2.30	2.50	2.30	0.130	0.705	0.473	0.538
Yolk index	43.74	44.65	46.82	45.99	45.65	0.935	0.253	0.116	0.155
Haugh unit	85.19	86.16	85.49	87.38	86.68	1.135	0.672	0.260	0.863
L*	2.449	1.117	2.520	1.813	1.501	0.7676	0.644	0.567	0.912
a*	0.873	0.606	0.561	0.613	0.599	0.4724	0.992	0.737	0.753
b*	31.96^b^	35.30^a^	34.74^a^	35.67^a^	34.49^a^	0.600	0.003	0.011	0.004

L* lightness, a* redness, b* yellowness, S.E.M.* standard error means, *L* linear effect, *Q* quadratic effect. ^a,b^Means with different superscripts in the same row were significantly different (*P* < 0.05)

Significant differences were found in the antioxidant capacity of the yolk when PCP was added as shown in Table 6. The TBARS method was used to evaluate the inhibition ability of PCP on lipid peroxidation. In this sense, feeding 0.4% PCP to laying quails caused a decrease in TBARS (*P* < 0.01) value (3.868 µmol MDA/kg) compared to the control (5.225 µmol MDA/kg). As PCP levels in the diet increased, TBARS value decreased. Contrary, the DPPH value increases as the dietary PCP levels increased (*P* < 0.01). Moreover, these values decreased further when dietary PCP levels exceeded 0.3%.

**Table 6:** Effect of addition of purple carrot powder in the diet on TBARs and DPPH of yolk in laying quails

Parameters	Purple carrot powder (%)					S.E.M*	P	L	Q
	0	0.1	0.2	0.3	0.4				
TBARS (µmol MDA/kg)	5.225^a^	4.745^ab^	4.574^abc^	4.207^bc^	3.868^c^	0.2488	0.016	0.001	0.934
DPPH (%)	3.792^b^	4.807^ab^	4.516^ab^	5.006^a^	5.263^a^	0.3241	0.049	0.003	0.438

*TBARS* thiobarbituric acid reactive substances, *MDA* malondialdehyde, *DPPH* 2,2-diphenyl-1-picrylhydrazyl, S.E.M.* standard error means, *L* linear effect, *Q* quadratic effect. ^a,b,c^Means with different superscripts in the same row were significantly different (*P* < 0.05)

## Discussion

Recent years have seen an increased demand for different varieties of carrots. Consequently, there has been a rise in the waste generated (Hammershøj et al., 2010; Yusuf et al., 2021). Considering its nutritional content, which is rich in bioactive substances (Poudyal et al., 2010; Yusuf et al., 2021), purple carrots offer an attractive possibility for animal feed (Hammershøj et al., 2010; Titcomb et al., 2019). In the current experiment, dietary treatments with PCP did not negatively affect quail development and egg production, making PCP a viable option to use as quails feed. Similar findings on these parameters have been reported when natural sources of carotenoids were added to the hens (de Souza et al., 2019; Maia et al., 2022; Pirgozliev et al., 2022; Yunitasari et al., 2022) and broiler (Wang et al., 2017; Csernus et al., 2020) diets. According to a previously published study (Pérez-Gálvez et al., 2020), carotenoids are an important source of bioactive compounds that play an important role in preventing certain diseases. Probably, the lack of development improvement of the animals related to the PCP diet could be because quails were in similar health conditions. The performance improvement, related to the carotenoid diet, is most obvious when the health condition of the animals is compromised (Nabi et al., 2018; Csernus et al., 2020) or during environmental stress conditions (Saeed et al., 2017; Yunitasari et al., 2022).

The damaged egg and egg-breaking strength were unaffected by PCP dietary, while differences were observed between experimental diets for eggshell weight and eggshell thickness. Eggshell weight and eggshell thickness were increased as PCP rose in the diet. According to the meta-analysis carried out by Yunitasari et al. (2022), the effect of natural sources of carotenoids on these parameters is uncertain. Pirgozliev et al. (2022) described that supplementation with stevia in hens’ diet not affected eggshell weight or eggshell thickness. Similarly, Panaite et al. (2021) showed that dietary supplementation with different natural sources of carotenoids (linseed, kapia pepper, sea buckthorn pomace, and carrot) did not affect the egg weight, eggshell weight, and eggshell-breaking strength. However, these authors described an improvement in eggshell thickness when carrot was added to the diet. Various aspects affect the thickness of the eggshell, including minerals such as calcium, magnesium, and phosphorus, being the main inorganic constituents (Olgun et al., 2022). Carrots are an abundant source of minerals, such as potassium, manganese, phosphorus, calcium, sodium, iron, and magnesium (Yusuf et al., 2021). The increased bioavailability of the minerals contained in the quail feed leads to high intestinal absorption and consequent deposition in the egg, enhancing these parameters. Moreover, internal quality parameters in terms of the albumen index, yolk index, and Haugh unit remain constant for all experimental diets. The findings are consistent with results described in previous studies (de Souza et al., 2019; Panaite et al., 2021; Pirgozliev et al., 2022). According to previous authors (de Souza et al., 2019; Olgun et al., 2022), albumen’s protein levels depend on the albumen’s protein content, which is determined by the feed intake. PCP dietary probably did not increase the protein content of the diet, nor losses on digestion processes. Therefore, these values are not expected to differ between dietary groups.

Yolk color is considered one of the most important attributes of egg quality, which plays an important role in consumer decisions (Kljak et al., 2021a). In the current research, PCP dietary improved the b* value (yellowness) which was consistent with the findings of Titcomb et al. (2019) and Panaite et al. (2021) when carrot leaves and carrot dried were added to the hens´ diet. Comparable outcomes were reported when different natural sources of carotenoids were included in avian diets. For example, Kljak et al. (2021a) described changes in yolk color when natural carotenoid sources (marigold, calendula, and basil plant) were added to the hens’ diet. Díaz-Gómez et al. (2017) showed higher yellowness scores in meat when broilers were fed with high-carotenoid maize, which is consistent with the findings of Wang et al. (2017). These authors reported that the addition of marigold extract to a broiler diet significantly increased the b* value of the thigh muscle and skin, suggesting that natural pigments were generally more effective than synthetic ones in enhancing the yellowness, making PCP a be a viable option as an alternative to synthetic egg colorants. Several factors influence carotenoid distribution from feed to egg synthesis, including accessibility, openness, fringe tissue interest, a carotenoid compound, carotenoid liking, and the ability of lipid vesicles to cross liver and egg layers (Yunitasari et al., 2022). Hammershøj et al. (2010) described those purple carrots had a high pigment ability due to the higher beta-carotene content which is greater than traditional carrots. The findings of the current experiment suggest a higher bioavailability of pigment compounds that would be assimilable in the small intestine (Yunitasari et al., 2022) by laying quails, as reflected by changes in yolk color.

Egg yolks contain a high-fat content, making them vulnerable to lipid oxidation (Pirgozliev et al., 2022), which causes the deterioration of the product and decreases storage time (Wang et al., 2017). However, hens can deposit antioxidants into their yolks as a result of their diet, which may protect lipids when eggs are stored (Kljak et al., 2021b). In the current research, increasing PCP content in the quails’ diet led to improved yolk antioxidant capacity, which is confirmed by the reduction in TBARS value and the rise in DPPH value. Similar to the current results, previous studies have demonstrated that the addition of natural sources of carotenoids in the diet can alleviate the adverse effects of oxidative stress on the eggs (Kljak et al., 2021a; Panaite et al., 2021) and the meat quality of broilers (Wang et al., 2017) resulting in a decrease in malondialdehyde (MDA) content, which is consistent with our findings. Purple carrots show a high anti-oxidant capacity due to their composition, such as total organic acid or polyphenolic contents (Yusuf et al., 2021). Lutein and zeaxanthin are important bioactive compounds of purple carrots and contain a range of unsaturated bonds. Thus, these compounds can scavenge reactive oxygen species, inhibiting the activity of oxygen free radicals and preventing reactive oxygen free radicals from destroying normal cells (Wang et al., 2017), which could explain the results obtained in the current research. Moreover, as proposed by Pirgozliev et al. (2022), these results would suggest that the PCP addition may reduce the susceptibility to lipid oxidation of the eggs during storage, enhancing their shelf life. The current result could lead to consumers choosing these eggs over others when purchasing eggs.

## Conclusions

The PCP feeding does not negatively affect the performance or egg production of laying quails, making this ingredient a viable option for reuse waste by-products. Moreover, PCP enhances some eggshell qualities such as weight or thickness and positively affects the b* value, which is an important attribute for the purchase decision. Additionally, dietary PCP improves the oxidative stability of the yolk. These results further suggest that PCP would be well absorbed in the small intestine and would subsequently be transferred to the quail egg.

## Data Availability

The authors declare that all the data and materials used in this study comply with field standards and available on demand.
